# What makes ‘hard decisions’ hard? Decision‐making in dyads of people living with Alzheimer’s disease and their care partners

**DOI:** 10.1002/alz.087665

**Published:** 2025-01-09

**Authors:** Emily A. Largent, Claire M. Erickson, Anjali Gupta, Melanie Kleid, Kristin Harkins, Shana D. Stites, Andrew H. Peterson, Jason Karlawish, Justin Clapp

**Affiliations:** ^1^ University of Pennsylvania, Philadelphia, PA USA; ^2^ Department of Medical Ethics and Health Policy, University of Pennsylvania, Philadelphia, PA USA; ^3^ Department of Psychiatry, Perelman School of Medicine, University of Pennsylvania, Philadelphia, PA USA; ^4^ George Mason University, Fairfax, VA USA

## Abstract

**Background:**

People living with Alzheimer’s disease and related dementias confront numerous decisions that affect their wellbeing, as well as that of their family members. Research demonstrates the importance of family involvement in such decision making, yet there is a lack of knowledge about how patients and families work together to make decisions and how families can best provide decisional support.

**Methods:**

Semi‐structured interviews were conducted separately with 15 patients diagnosed with mild cognitive impairment (MCI) or mild dementia, identified through a National Institute on Aging‐funded Alzheimer’s Disease Research Center, and 14 care partners. As part of a study of decisional support, interviewees were asked to describe a hard decision they had faced. Data analysis was guided by a constructivist grounded theory approach and consisted of iterative rounds of coding with checks for intercoder reliability.

**Results:**

Patients and care partners most frequently identified the patient no longer driving as a hard decision; other hard decisions included changing the patient’s living arrangements or surrendering responsibility for their finances. Though interviewed separately, dyad members frequently identified the same hard decision. Hard decisions were characterized by tension between the patient’s independence or identity and the safety of the patient or others; the tension was nearly always resolved in favor of safety, particularly safety of others. Despite characterizing these as “decisions,” patients often felt they had no meaningful choice and implied that the outcome was inevitable. Consistent with these findings, both patients and care partners typically indicated that care partners initiated these decisions and denied engaging in substantive deliberation before enacting changes. Patients and care partners described hard decisions as emotionally difficult because they were confronted with the progression of the patient’s disease. Even as they acknowledged the importance of safety, patients grieved their loss of independence and diminished sense of self. Care partners expressed empathy for the patient’s losses and grieved alterations to the relationship.

**Conclusions:**

Hard decisions are associated with undesirable outcomes more than with difficult decision‐making processes. When disease progression necessitates change, patients and care partners grieve even as they acknowledge change is appropriate. They may benefit from others acknowledging this grief.

